# Supplementary figures and images for: Oligopaint DNA FISH reveals telomere-based meiotic pairing dynamics in the silkworm, Bombyx mori
Source: PLoS Genet. 2021 Jul 28;17(7):e1009700. doi: 10.1371/journal.pgen.1009700 (PMC8351950; doi:10.1371/journal.pgen.1009700)

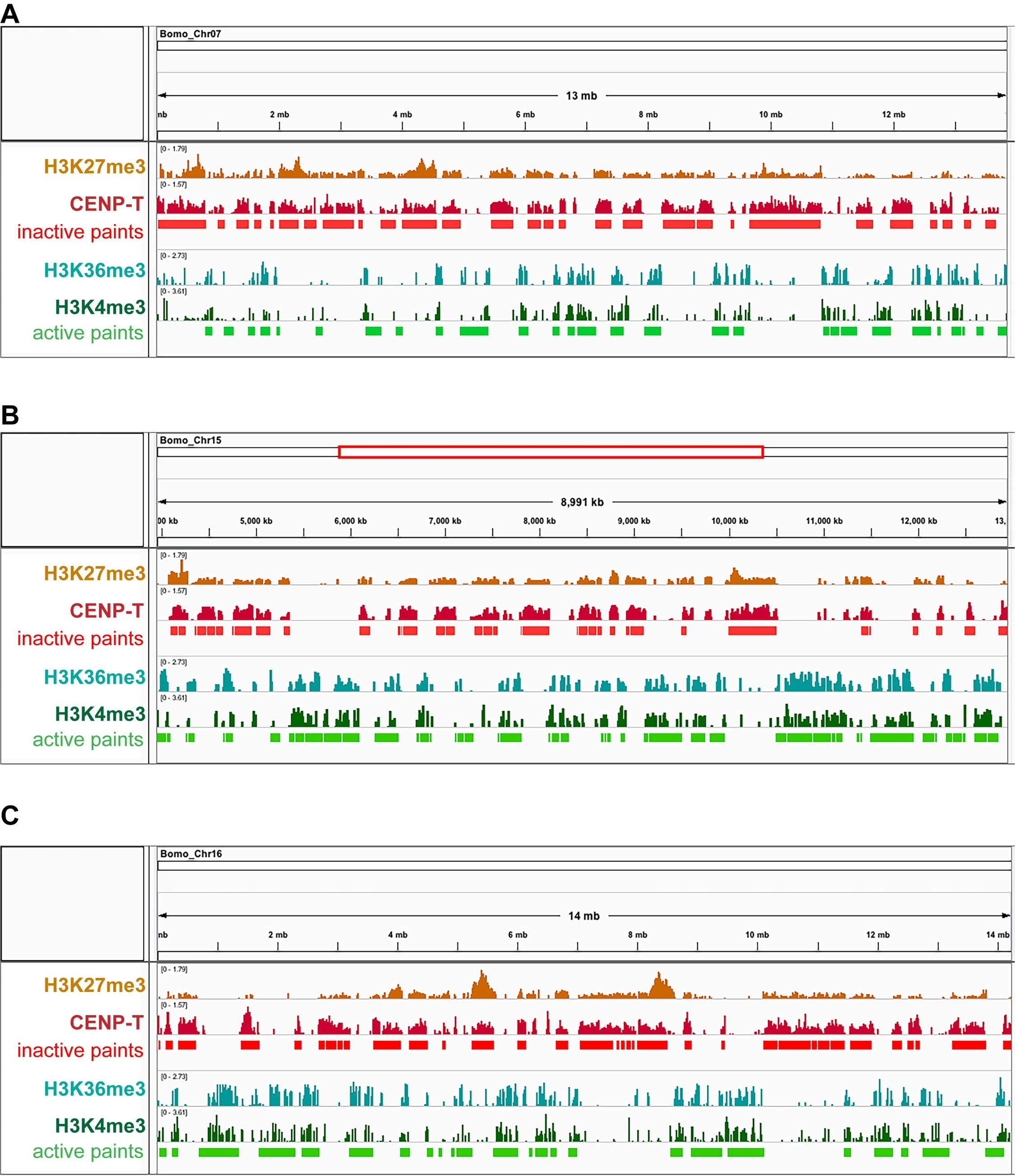

Supplement: S1 Fig — Screenshots of ChIP-seq data used to design active/inactive chromosome paints. Inactive: H3K27me3 (orange), Centromere Protein T (CENP-T; dark red). Called inactive paint domains shown in bright red. Active: H3K36me3 (teal), H3K4me3 (dark green). Called active paint domains shown in bright green. ChIP-seq data were previously published (see Materials and Methods). Chromosome 7 is shown in A, part of chromosome 15 is shown in B (coordinates Chr15:3,900,000–12,891,000), and chromosome 16 is shown in C. (TIF) [file pgen.1009700.s001.tif]

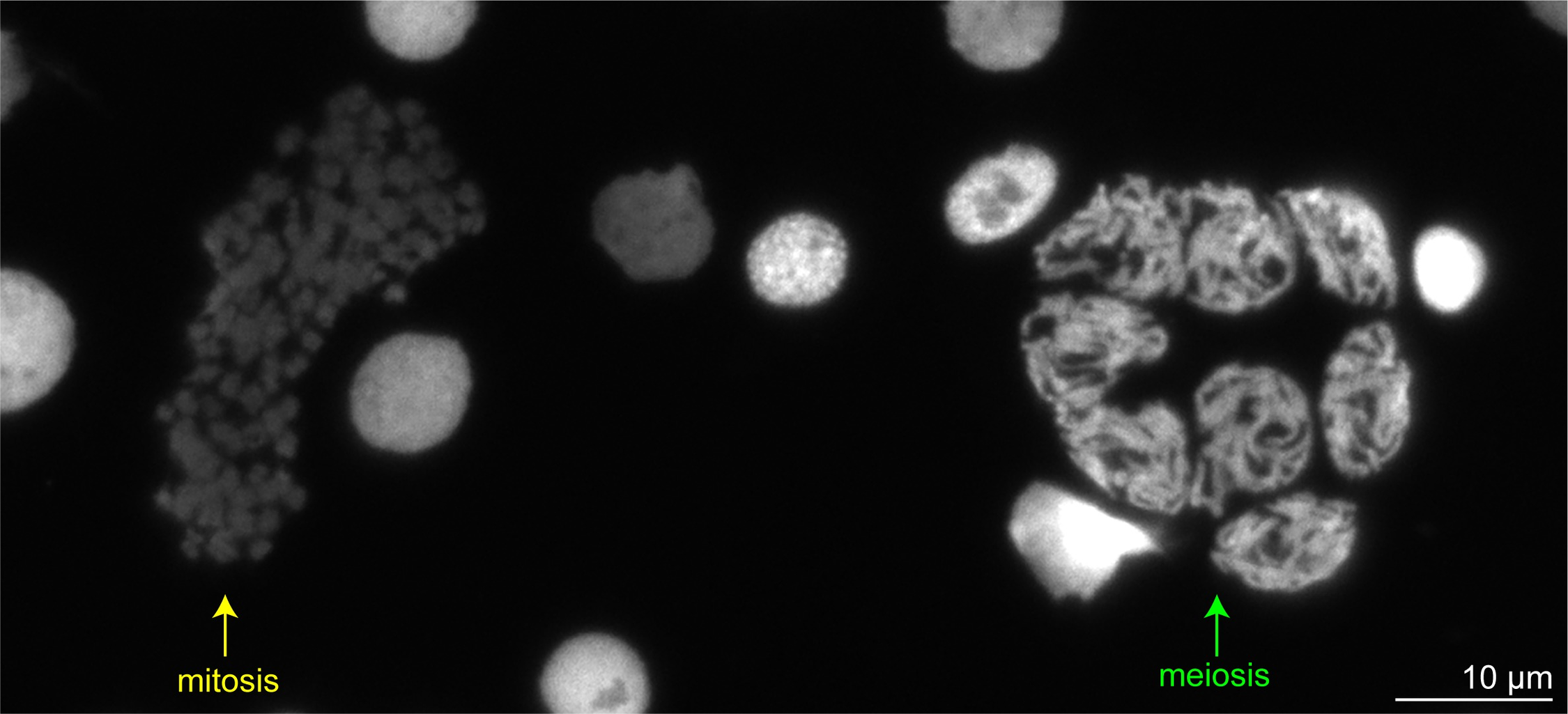

Supplement: S2 Fig — Representative image showing a cluster of mitotic chromosomes (yellow arrow, left) and a cluster of meiotic prophase I cells (green arrow, right) labeled with DAPI. (TIF) [file pgen.1009700.s002.tif]

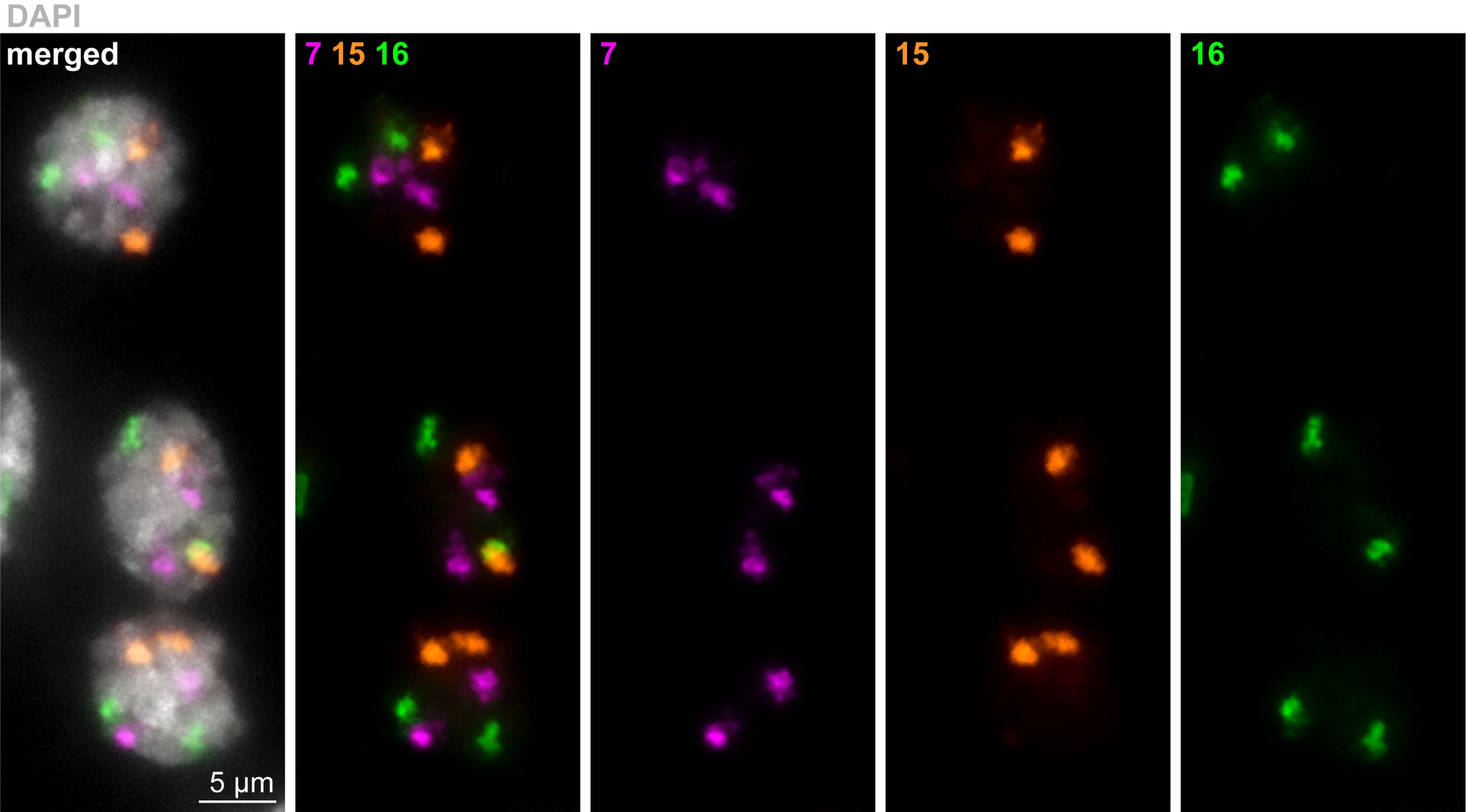

Supplement: S3 Fig — Three nuclei (DAPI in gray) labeled with Oligopaints for ch7 (magenta), ch15 (orange), and ch16 (green) in somatic cells from late 4th instar larval testes gonad squashes. Two signals per nucleus indicate unpaired homologs. (TIF) [file pgen.1009700.s003.tif]

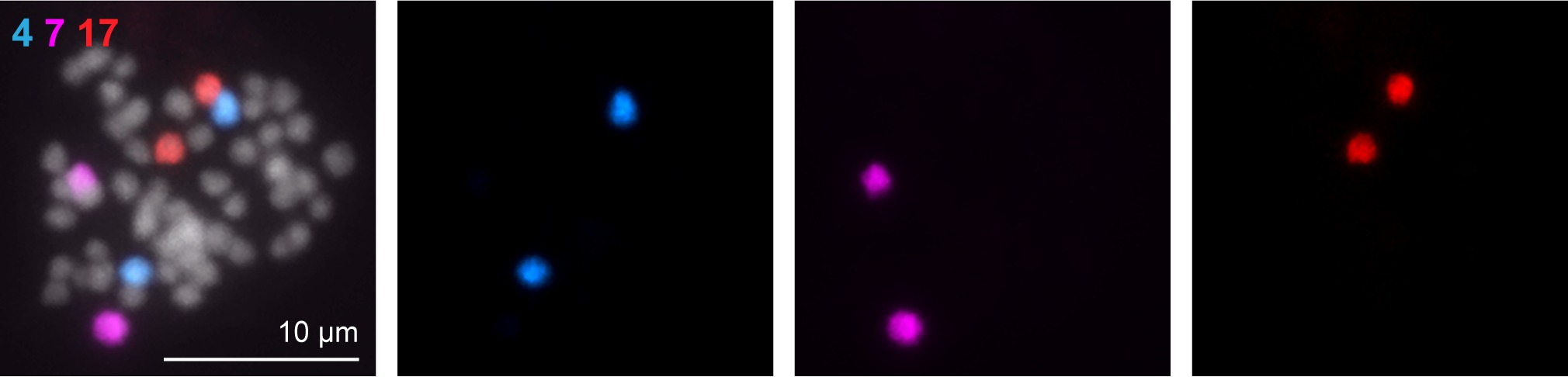

Supplement: S4 Fig — Representative mitotic cell labeled with whole chromosome paints for ch4 (blue), ch7 (magenta) and ch17 (red). (TIF) [file pgen.1009700.s004.tif]

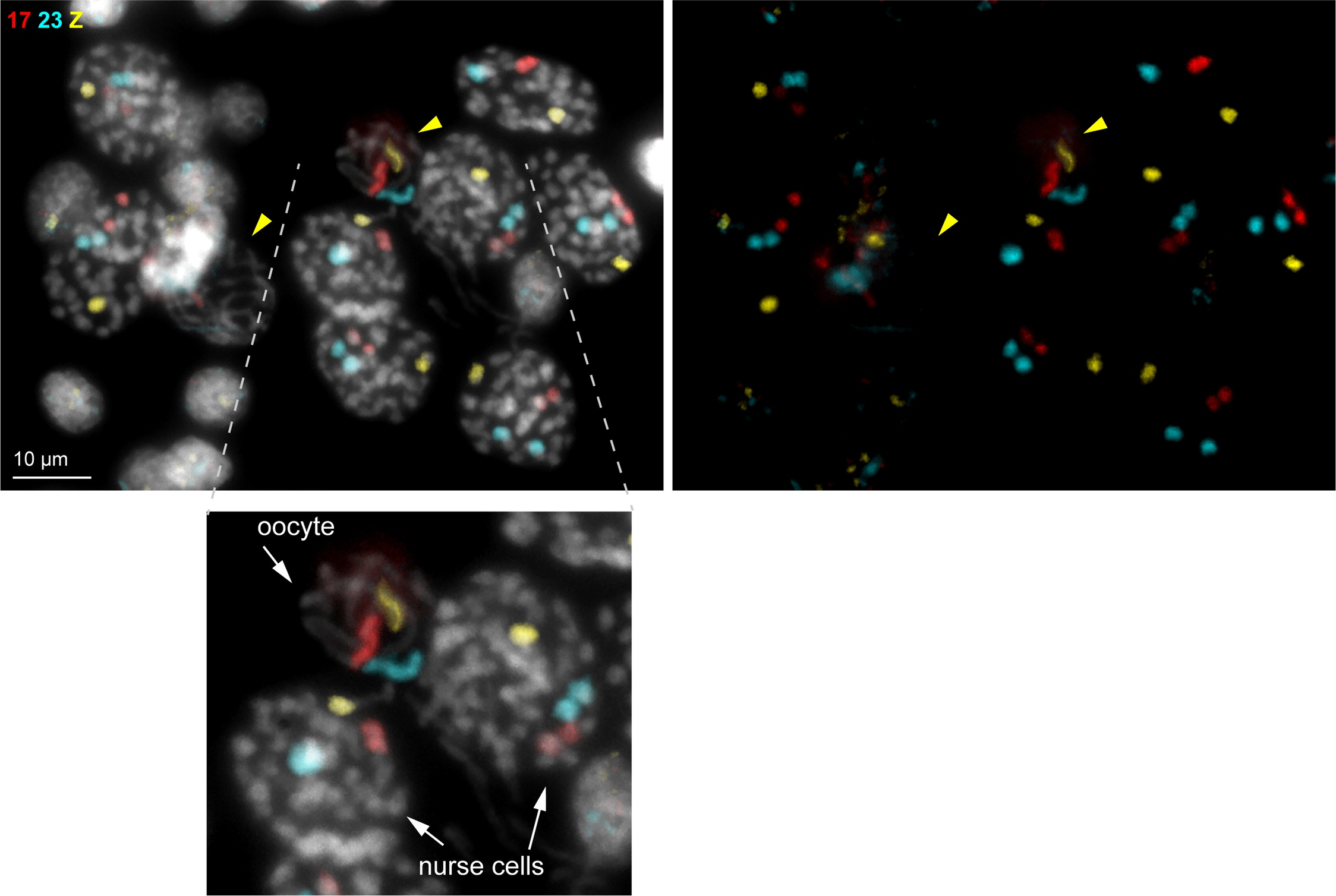

Supplement: S5 Fig — Top: Representative field from early 5th instar larval ovary squash labeled with whole chromosome paints for ch17 (red), ch23 (cyan), and chZ (yellow). Left, merged with DAPI, right, paints alone. Yellow arrowheads indicate early differentiating oocyte cells. Note: female B. mori are heterogametic and harbor a single Z and a single W chromosome (compared to two copies of ch17 and ch23). Bottom: zoom showing early differentiating oocyte and nurse cells, as indicated. As both oocytes and nurse cells undergo homolog pairing in early meiotic prophase, homologs remain in close proximity in all early differentiating nurse cells. (TIF) [file pgen.1009700.s005.tif]

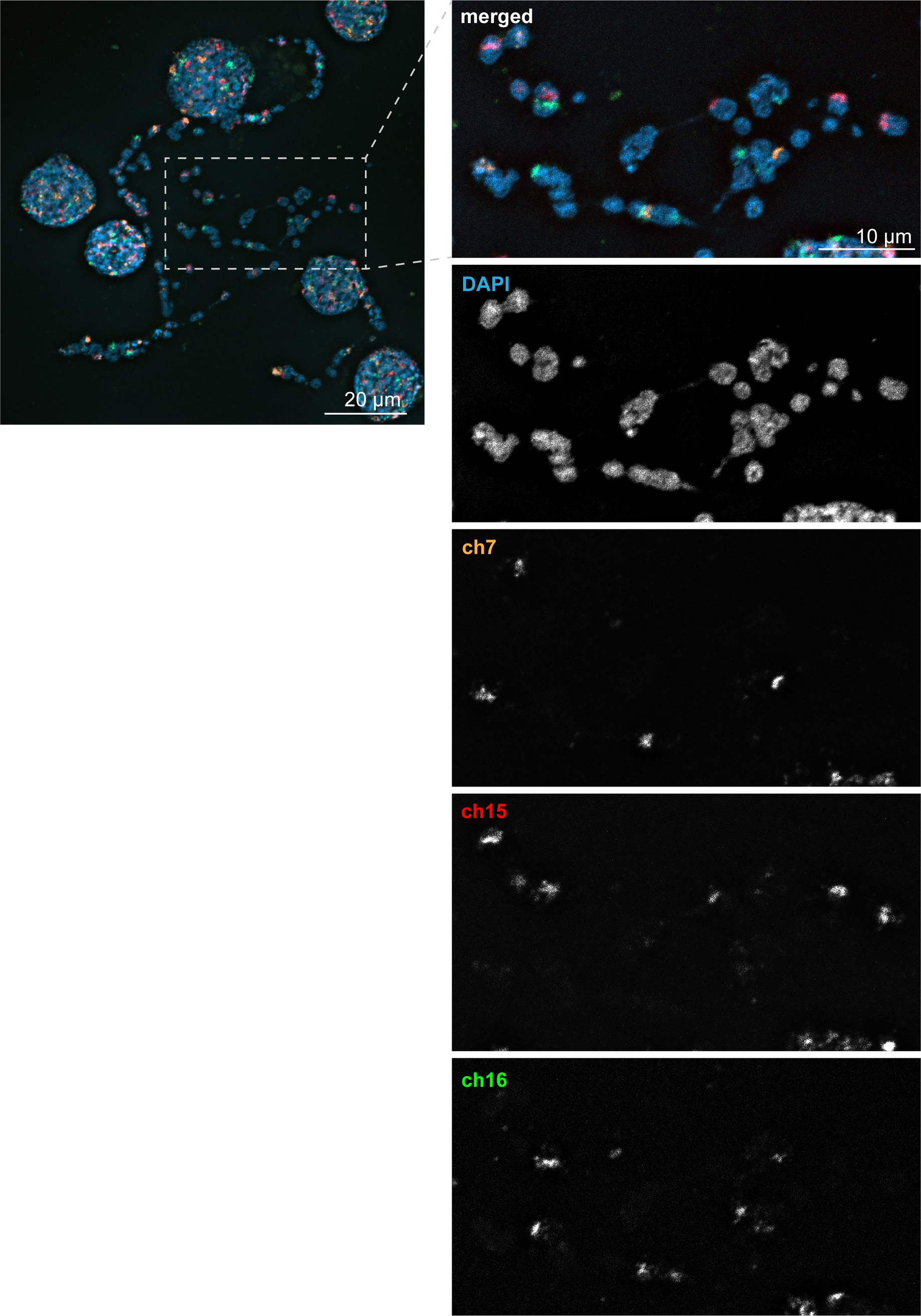

Supplement: S6 Fig — Left: representative mitotic chromosome spread from BmN4 cultured cells labeled with whole chromosome paints for ch7 (orange), ch15 (red), and ch16 (green). Gray dashed box indicates zoom shown to the right. No entire chromosomes are labeled in the cell line, and instead, several chromosomes are partially labeled with each paint, indicating a large number of translocations in this cell line compared to animals. (TIF) [file pgen.1009700.s006.tif]

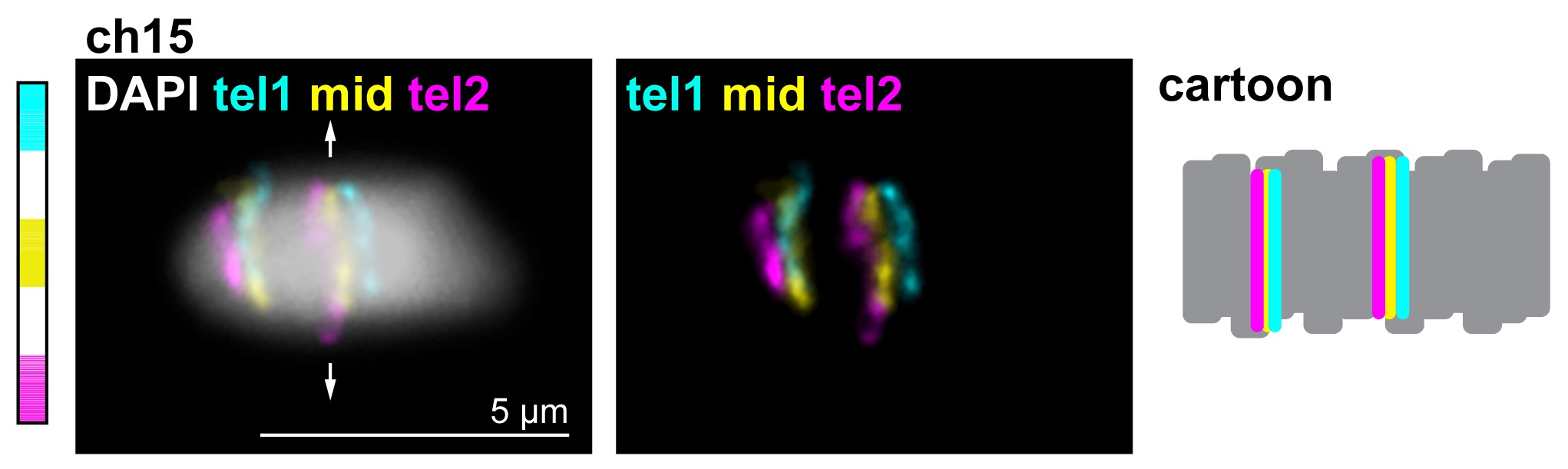

Supplement: S7 Fig — Left: schematic of ch15 with stripe paints. Middle two panels: Representative nucleus at metaphase of mitosis labeled with ch15 stripe paints. Arrows indicate the direction of the spindle poles. Right: Cartoon representation of nucleus in middle panels. (TIF) [file pgen.1009700.s007.tif]

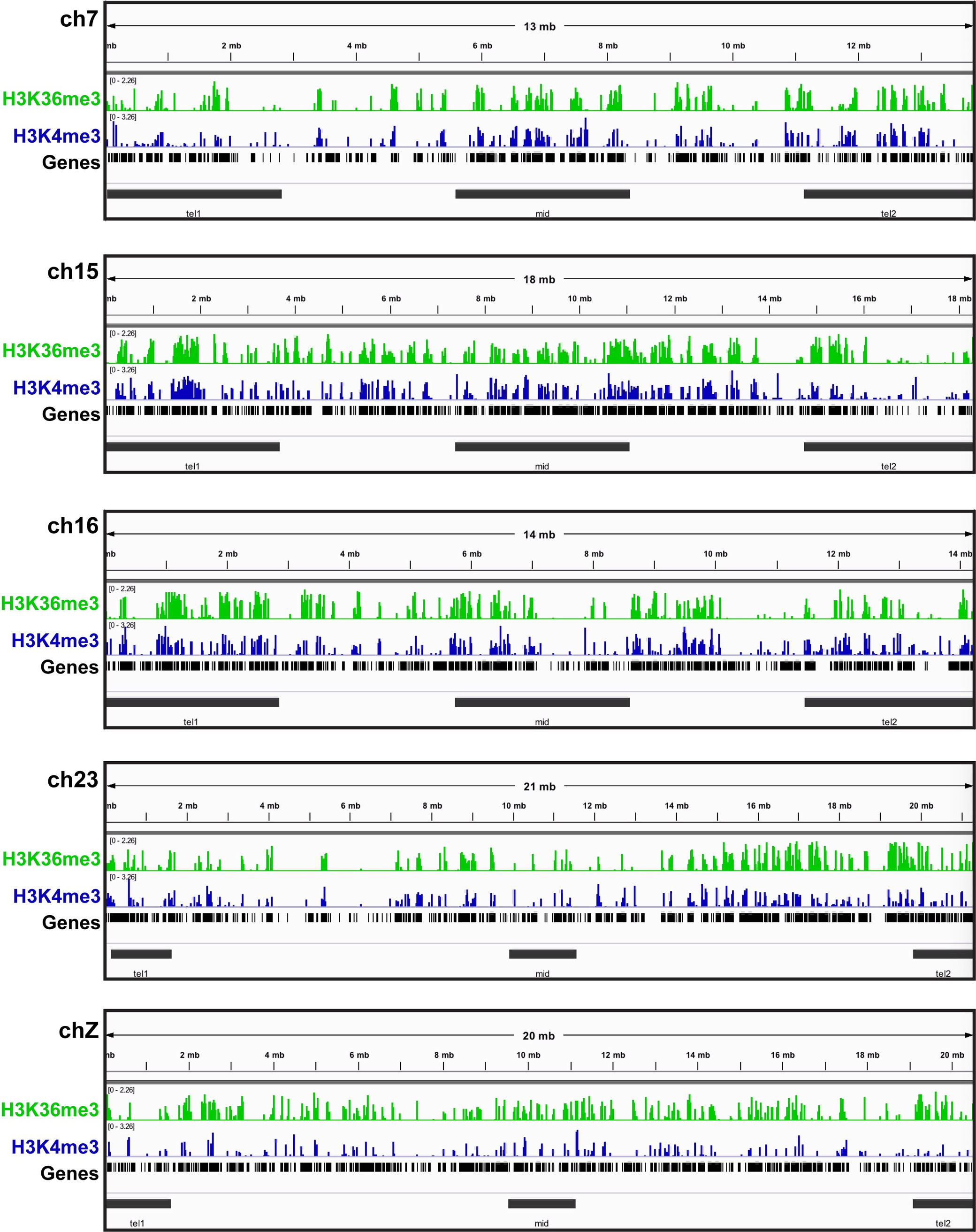

Supplement: S8 Fig — Screenshots of genome browser views of ch7, 15, 16, 23, and Z. Top: ChIP-seq profiles for active histone modifications from BmN4 cells: H3K36me3 (green), H3K4me3 (blue). Middle: gene positions. Bottom: tel1, mid, and tel2 Oligopaint domains. Note: ch15 tel2 is depleted of genes and active histone marks compared to tel1, while ch23 tel2 is enriched for active marks and genes compared to tel1. (TIF) [file pgen.1009700.s008.tif]

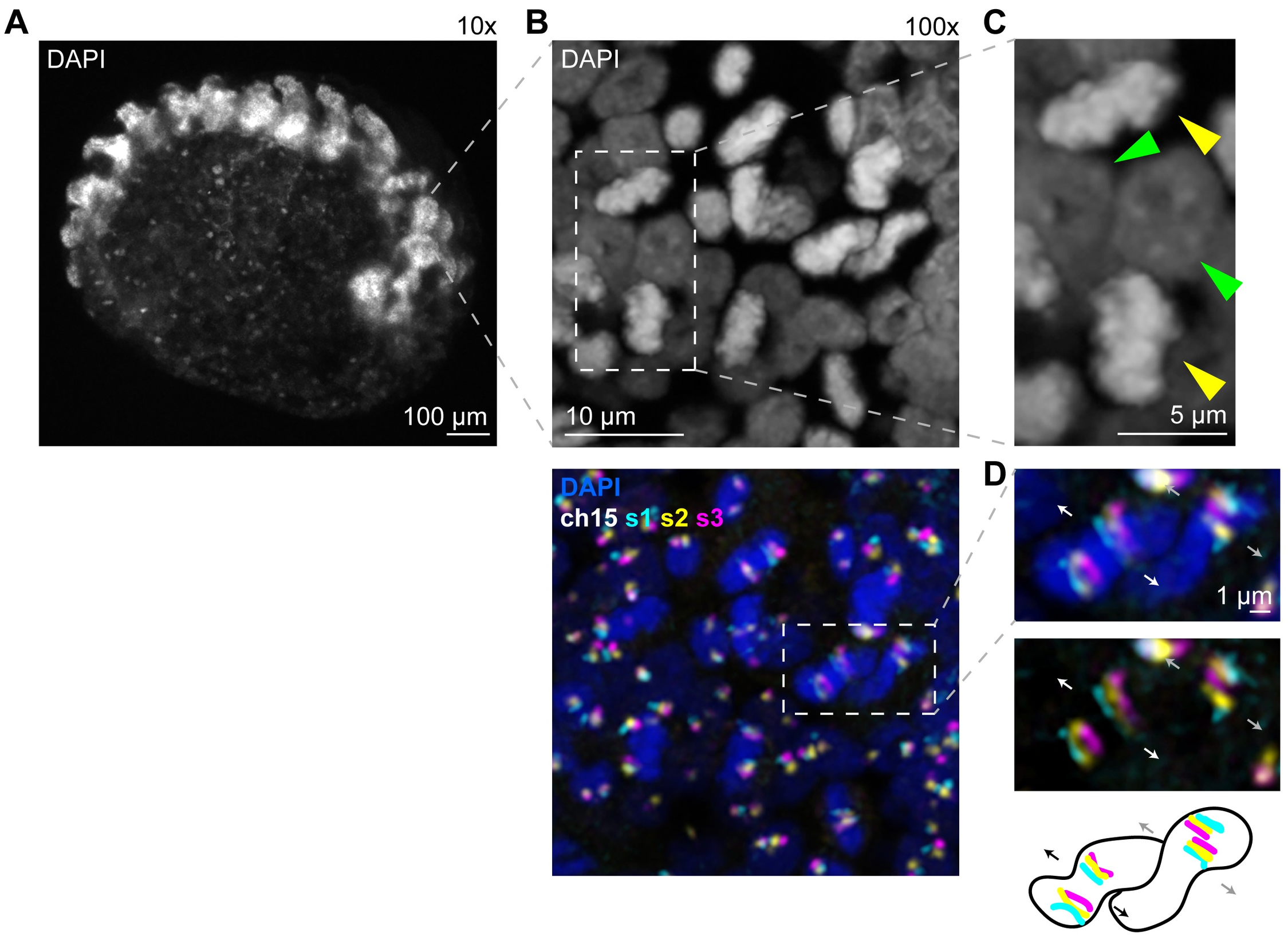

Supplement: S9 Fig — (A) Whole-mount embryo stained with DAPI imaged at 10x. (B) 100x image of whole-mount embryo stained with DAPI (top) and labeled with ch15 stripe paints (bottom) showing representative mitotic and interphase somatic cells. Note: both 10x and 100x images show Z-projections of multiple Z-stacks, with the 100x being a sub-stack of the 10x. (C) Zoom of B (top). Yellow arrow heads indicate mitotic cells, green arrow heads indicate interphase cells. (D) Zoom of B (bottom), showing two mitotic cells labeled with ch15 stripe paints and DAPI stain (top) or only stripe paints (middle). White and gray arrows indicate direction of spindle poles. Bottom, cartoon schematic, with the black outline representing the border of the DAPI stain. Black and gray arrows indicate direction of spindle poles. (TIF) [file pgen.1009700.s009.tif]

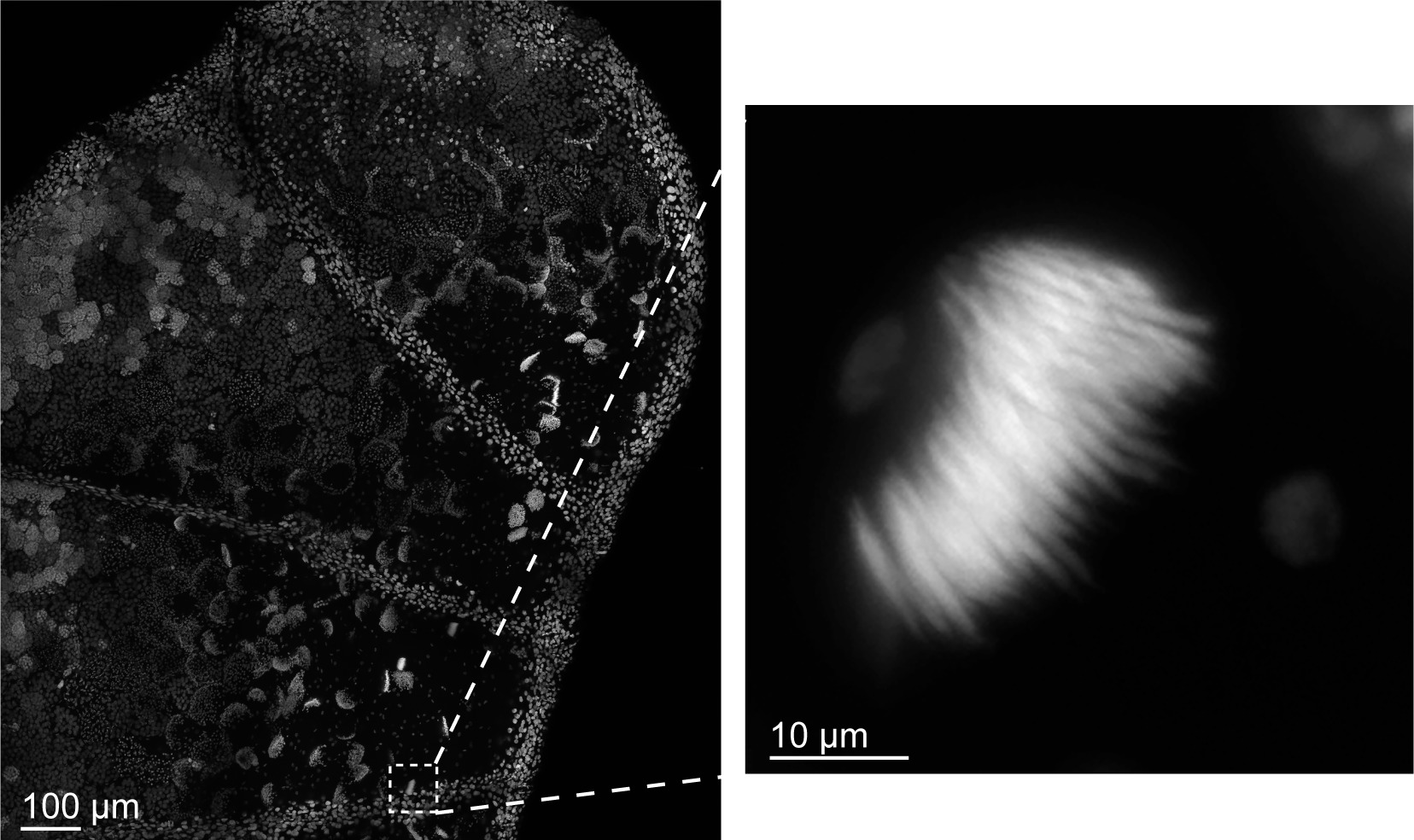

Supplement: S10 Fig — Left: 10x image of whole mount larval testis stained with DAPI. White box indicates zoom shown to the right. Right: zoom of mature eupyrene sperm bundle labeled with DAPI. (TIF) [file pgen.1009700.s010.tif]

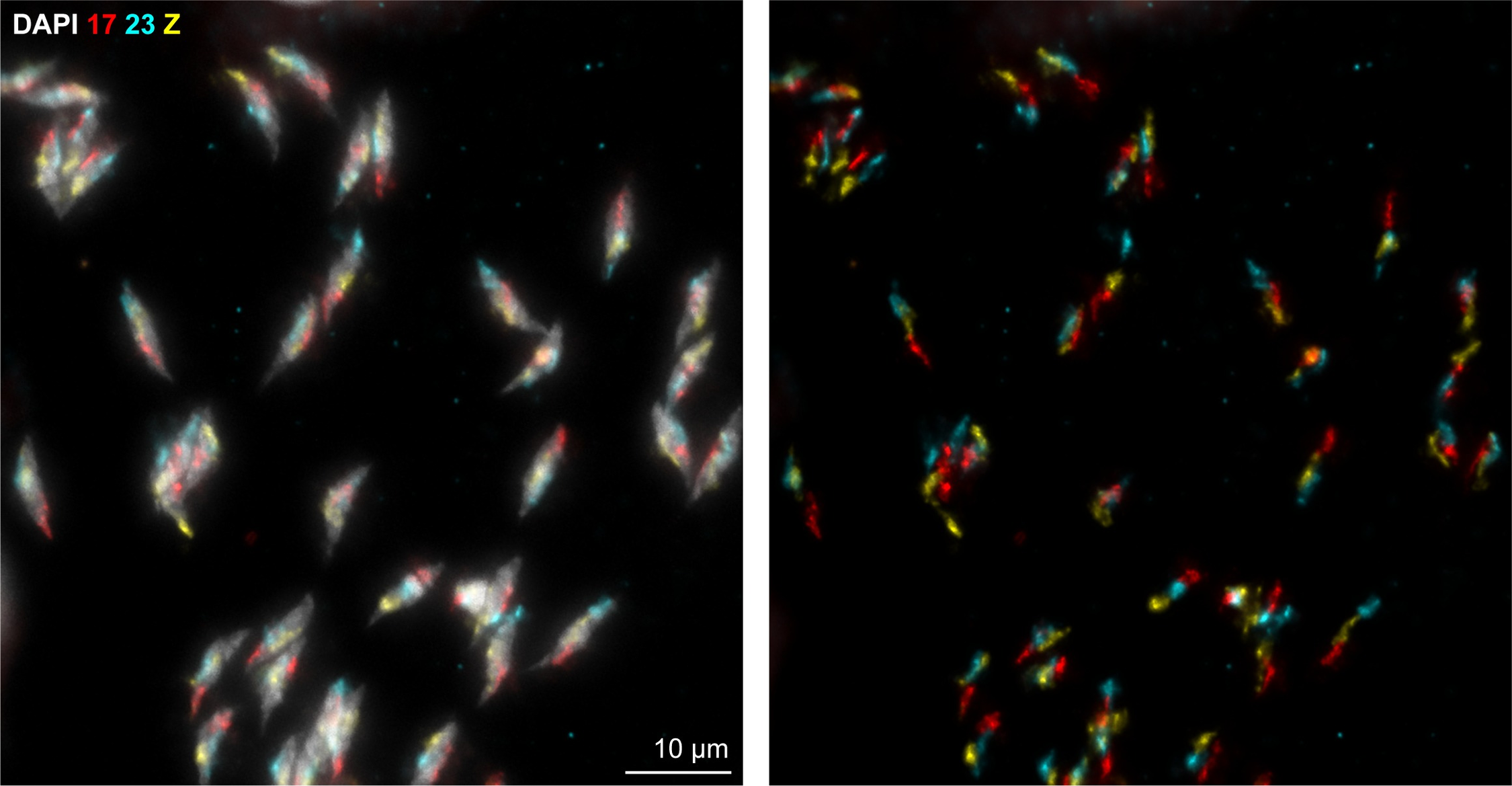

Supplement: S11 Fig — Oligopaints labeling chromosomes 17 (red), 23 (cyan) and Z (yellow) in mature sperm from a 5th instar larval testis squash. DAPI is shown in gray. (TIF) [file pgen.1009700.s011.tif]

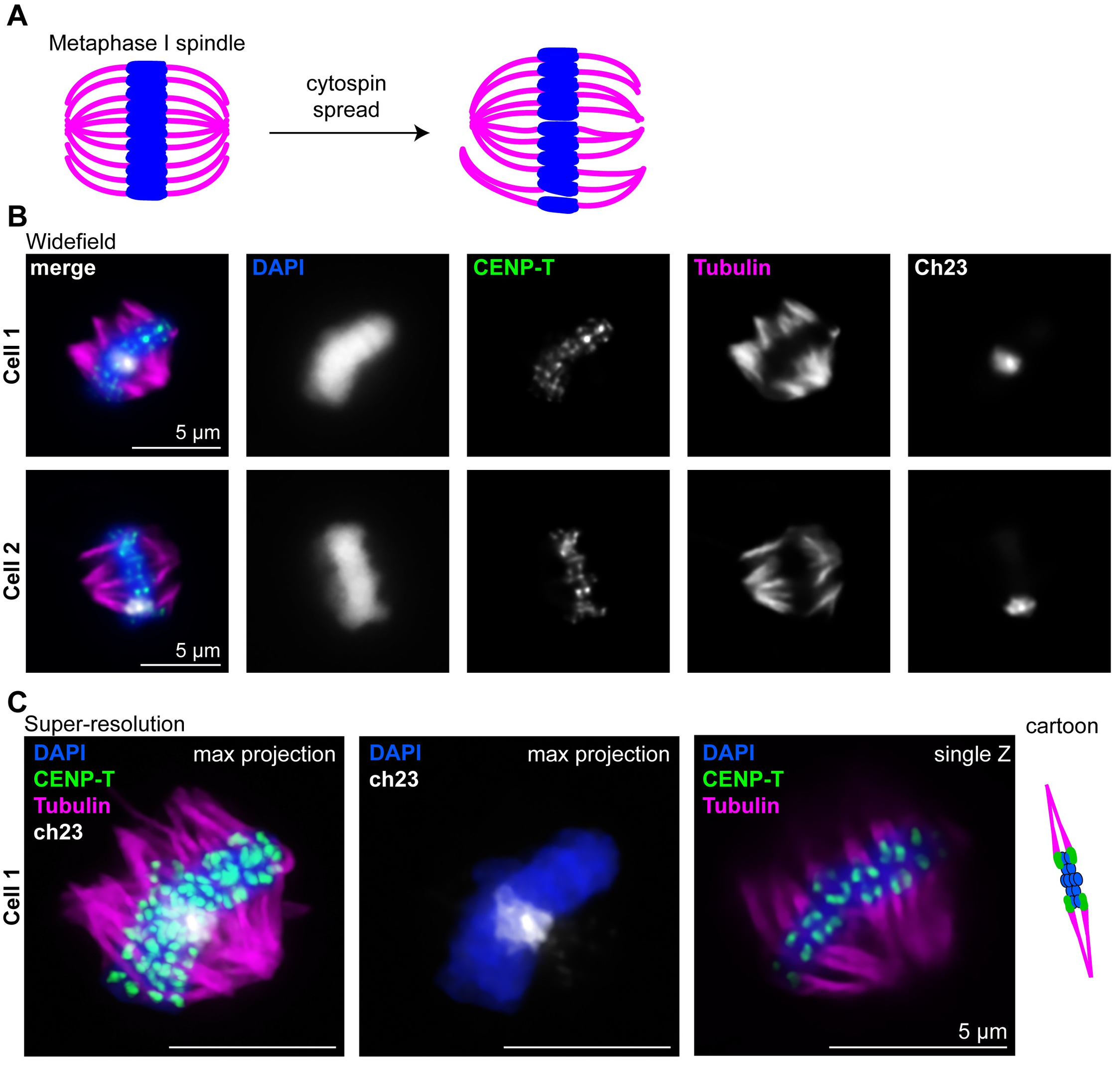

Supplement: S12 Fig — (A) Schematic of intact metaphase I spindle and aligned chromosomes from larval testes and broken spindle with spread chromosomes after cytocentrifugation. (B) Widefield images from dissociated testes cytospreads showing representative cells (two) with CENP-T localization on meiotic chromosomes. Images show Z-projections of 5–10 Z stacks. Scale bars = 5 μm. (C) Left: Super-resolution image images showing CENP-T binding to a metaphase I cell. Right: cartoon schematic of a single chromosome (blue) with CENP-T kinetochores in green and microtubules (anti-Tubulin staining) in magenta. (TIF) [file pgen.1009700.s012.tif]

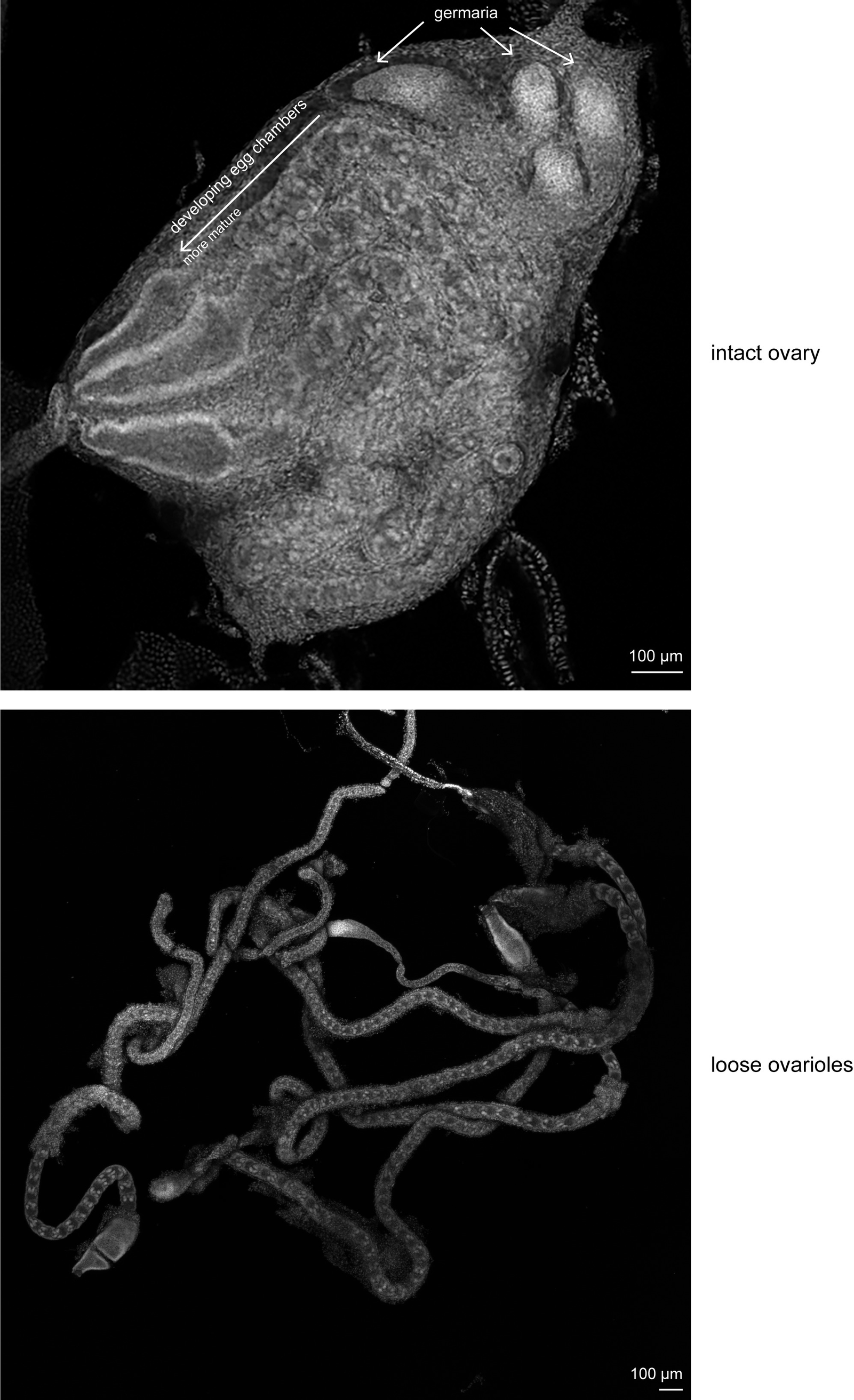

Supplement: S13 Fig — DAPI staining on whole mount 5th instar larval ovaries. Intact ovary is shown on top and below are loose ovarioles. (TIF) [file pgen.1009700.s013.tif]

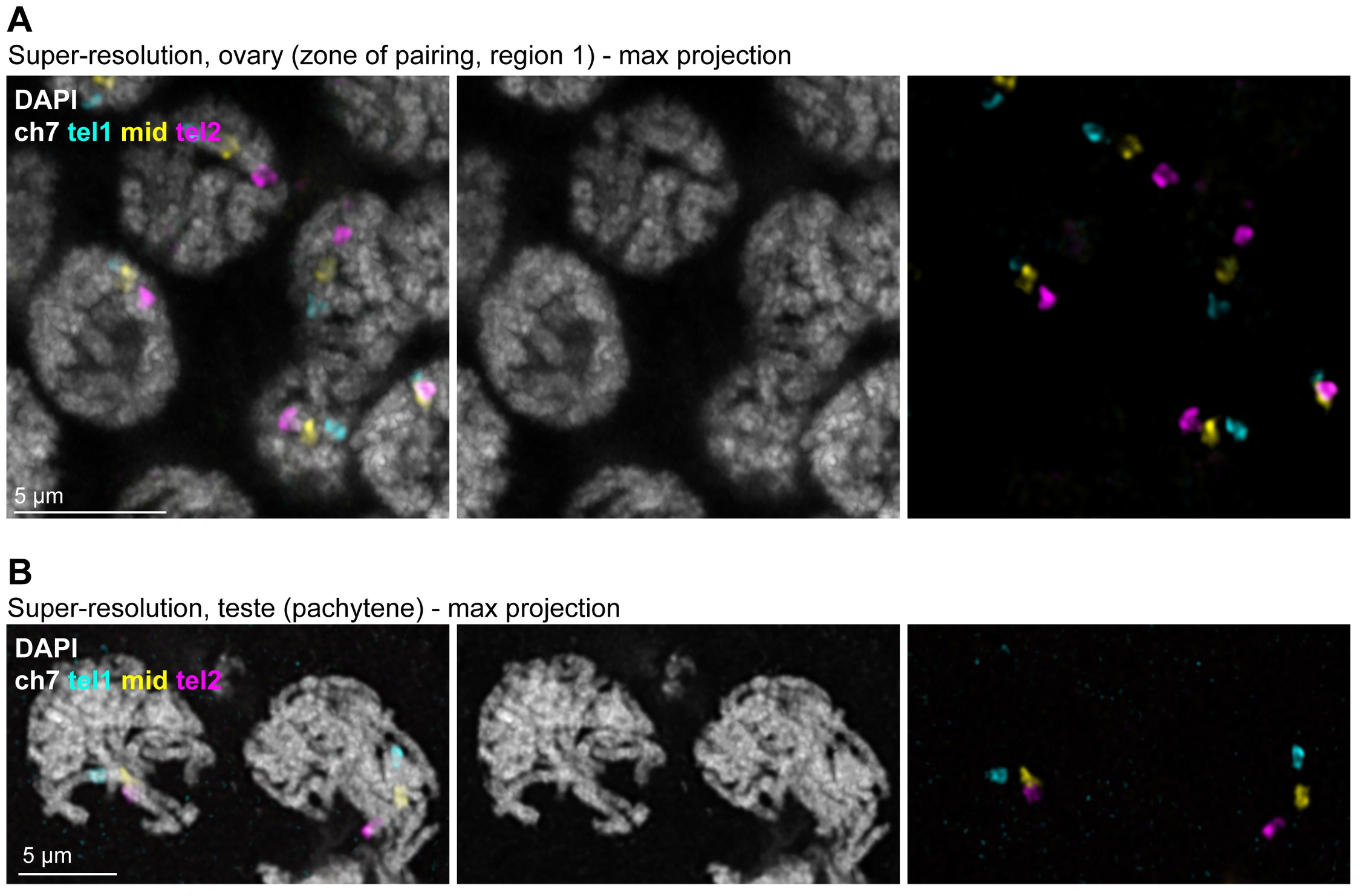

Supplement: S14 Fig — (A) Super-resolution image of a representative field of cells in the zone of pairing (region 1) in whole mount larval ovaries labeled with ch7 stripe paints. (B) Super-resolution image of two representative pachytene cells in larval testes squashes (to maximize the possibility of observing space between the homologs) labeled with ch7 stripe paints. (TIF) [file pgen.1009700.s014.tif]
