# Supplementary material for: Oligopaint DNA FISH reveals telomere-based meiotic pairing dynamics in the silkworm, Bombyx mori
Source: PLoS Genet. 2021 Jul 28;17(7):e1009700. doi: 10.1371/journal.pgen.1009700 (PMC8351950; doi:10.1371/journal.pgen.1009700)
Supplement: Attachment — Submitted filename: Rosin et al - response to reviewers.pdf [file pgen.1009700.s017.pdf]

We thank all the reviewers for taking the time to provide valuable and constructive feedback on our manuscript. We have addressed each of their points through changes in the text, figures, higher resolution imaging, and the addition of further data analyses and quantification. As a result, we feel that the manuscript has greatly improved with respect to novel insights into *B. mori* meiosis and homolog pairing in both males and females. We have thus also changed the title of the manuscript to more accurately reflect the major findings therein. Below we provide responses to the reviewers' points below in blue font.

Reviewer #1:
In Rosin et al. the authors use Oligopaints to look at chromosome pairing during female and male meiosis in the silkmoth Bombyx mori. Using the Oligopaints to both full chromosomes and specific sections of the chromosomes showed that chromosomes undergo progressive pairing until fully paired along their lengths. In males the one end of the chromosome becomes unpaired while the other end remains associated when chromosomes become aligned. The authors suggest the unpaired end acts as a kinetochore to segregate the chromosomes at the first division.

The imaging beautifully shows the pairing of the chromosomes, but the authors need to temper some of their claims before publication. The authors make several assumptions that need to be demonstrated in the manuscript or those claims need to be softened.

For example the authors claim in males the configuration they show when chromosomes are lined up are in metaphase I and the chromosomes are aligned towards the spindle poles but no images include a spindle. Therefore the data does not CLEARLY demonstrate that telomeric regions face poleward as stated in several places. The authors are assuming that a spindle is present and orients in that direction. Either spindle images with DNA at the same stage/ configuration need to be supplied or claim changed to "most likely" faces the spindle pole.

We thank the reviewer for pointing out this issue. We have added some images of tubulin co-staining with DNA FISH to the supplement (new Figure S12).

It is claimed that pairing is maintained after the loss of the central region on page 22 and after the SC has reformed in other locations. With no immunofluorescence images of the SC shown that are taken at the same stages as oligopaints or taken with the oligopaints (the better experiment) the authors can only speculate about the status of the SC at the stages examined.

We agree with the reviewer, but unfortunately we do not yet have any antibodies for SC components in *B. mori*. Therefore, we have tempered this and other statements made about SC transformation to state "reported" loss or "thought to be" remodeled (for example, lines 106-109). We hope this satisfies the reviewer's concerns.

The authors need to be clearer in text, not just methods, the stages were classified based only on DAPI. Staging assessments were not collaborated using stage specific antibodies to the SC, determination markers, or stage specific structures as is standard in pairing studies in other meiotic systems and authors should be clear that the staging are estimated. This is particularly important if the authors did spread experiments, since in spreads temporal information is normally lost.

We have added a statement (lines 172-173) to the main text describing our method for staging cells, as well as more specific details throughout that entire paragraph.

There is also confusion on whether the authors did chromosome squashes or spreads. The terms seem to be used interchangeably for the images in Figure 2 but the techniques are different with different results. In spreads tissues and cells are disrupted and the free nuclei are spread on a slide, which is why temporal information is lost. In squashes a whole tissue is flattened onto a slide/ coverslip. While the tissue is distorted and nuclei can burst in the squashing process some temporal information is retained since cell burst in relation to their neighboring cells.

We thank the reviewer for pointing out this discrepancy, and we have clarified our approach both in the main text (lines 164-167) and in the methods and revised the text to refer to the approach as "squashes" where appropriate.

In the zone of pairing how does the author know which cells are the meiotic cells compared to somatic cells without a differentiation marker?

While we cannot definitively distinguish between very early meiotic and somatic cells, we used chromosome morphology by FISH to differentiate between early meiotic cells and somatic cells (long, linear chromosomes upon meiotic entry versus small, spherical chromosomes that are unpaired in somatic cells.) We have clarified this in the main text (line 181-182) as well as to the methods.

Minor notes
Line 86 should read segregation OF meiotic chromosomes and Line 239 needs a reference to who previously hypothesized the configuration in Bombyx.

We thank the reviewer for bringing these errors to our attention, and we have corrected them.

Reviewer #2:
This paper describes results the Authors have obtained applying oligo-paint technology to Bombyx mori meiosis. The results show that the meiotic chromosome organization and behavior can be studied using these techniques. The most interesting and significant results are in Figure 4 that show that one end of each bivalent ends can lead the chromosomes towards the poles. This is important because B. mori chromosomes lack localized centromeres in mitosis. Like C. elegans, these results show that one end of the chromosome assumes the role of a centromere by leading the way to the pole meiosis I pole. The fact that either end can assume this activity suggests this is like C. elegans, and a mechanism exists to restrict kinetochore-like activity to one end, like the position of the crossover. This makes the females extra interesting, because these chromosomes segregate without crossovers.

While these results are interesting, they mostly demonstrate the techniques and do not go into much depth. This is not to say these results are not important, only that they mostly represent a technological achievement. The paper could be improved with more rigor and quantitation, which would allow others to know what to expect when doing experiments in this system. A particularly valuable feature of both germlines is the array of meiotic stages that can be observed. However, it is difficult using the information in this paper to appreciate the organization of the germlines. Furthermore, almost all data is present without any quantitation.

For example, how many oocytes or sperm are observed at each stage, and how many show the characteristics being reported? Some examples are below. By addressing these and the following comments, the authors may increase the accessibility and rigor of these results.

We thank the reviewer for their constructive feedback. We have added quantification to all figures where appropriate, including Figure 2 to show the proportion of cells in each stage of meiotic prophase.

1) In Figures 2, 5 and 6, the organization of the germlines could be hard to understand. For example, in Figure 2F, why is diplotene between zygotene and pachytene. Schematics would the reader understand the relationship between Figure 2 and Figure 5A-C. Thus, for both the male and female germlines, a schematic showing the organization of the meiotic stages should be added. When showing meiotic metaphase or mitosis images, it would help to indicate the axis of the spindle (because there is no tubulin or spindle pole labels). Figure 6, line 342: how are the oocytes identified.

We thank the reviewer for pointing out the lack of clarity in these figures. We have better described our meiotic spread protocol in both the main text and methods, explaining more clearly how spatial and temporal information are not necessarily retained during this process (lines 164-167). We hope this clarifies, for example, why diplotene cells are positioned on the slide between zygotene and pachytene. We have also moved the schematic for the male germline to the main figures (Figure 6), and we have added arrows indicating the spindle axis to all figures where appropriate. Finally, we have added a description for how oocytes are identified to the legend of Figure 7 (lines 573-574).

2) Line 89 and 386-388 – the idea the microtubules directly penetrate the chromatin should be stated with caution for two reasons. First, the lack of a CENP-A homolog, which is a centromere protein, does not mean there are no kinetochores. Second, kinetochore proteins are present on the chromosomes in meiosis of holocentric organisms like C. elegans, and they have an important role in meiotic chromosome segregation.

We have removed this statement about microtubules penetrating directly into chromosome ends and improved clarity regarding the finding that while *C. elegans* meiosis is independent of CENP-A, it is not independent of kinetochores (lines 36-37, 99-101, and 408-409).

3) While “elimination chromatin” is used in prior publications, it is a confusing term and the authors should use it with some explanation. Is chromatin actually eliminated? What is it actually referring to.

We have completely removed the term “elimination chromatin” from the text and instead refer to this phenomenon as “modified SC” or “SC transformation”.

4) Line 165: Describe in more detail how partially paired chromosomes are observed and define how often they are observed.

We have expanded this section to include more details and have quantified the different partially paired configurations we observed (starting in line 216 and new Figures 4 and 5).

4) Figure S3 is important, and part of it could be a main figure with quantitation. This could replace some redundancy in existing regular figures. For example, some figures (eg. #2) show the same thing with different probes. Figures 3 and 4 show similar data, with 4 showing more stages and detail. Seeing the

DAPI channel in 4A is not helpful and could be deleted and 4A,B,C show much the same thing.

We thank the reviewer for this suggestion, and we have added quantification and moved old Figure S3 to a main figure in the manuscript (new Figure 4).

5) Figure S5 – some images look like there is evidence of pairing in nurse cells. Please quantify.

As both nurse cell-destined germ cells and oocyte-destined germ cells pair at the beginning of female meiosis, all homologs in the early nurse cells are either directly juxtaposed or in close proximity. We have clarified this point in the text (lines 185-188).

6) Why is Figure S7 included or the text from lines 178-183. Explain the significance of this data or delete.

We have expanded our explanation for why we included the data from the ovary-derived cell line (lines 188-193).

7) Lines 212-213 – this description is confusing because it is written that the chromosomes were divided into 5 or 13 stripes. But the rest of the discussion seems to concern the 5 stripes (first, middle, last) and not more reference to experiments with 13.

We have reworded this section for increased clarity, removing the number of stripes that are unlabeled for clarity and renaming the labeled stripe domains as “telomere 1 (tel1)”, “middle (mid)”, or “telomere 2 (tel2)” (lines 201-205).

8) Line 238 “previously hypothesized” needs a reference.

Thank you for pointing this out; we have added the reference.

9) Figure 4B: Could the authors could come up with more descriptive or useful terms for S1, S2, S3. Furthermore, the cartoon schematic is not accurate – it implies and X-form structure which is not obvious in the images.

We thank the reviewer for pointing out this inconsistency between the microscopy and the cartoons. Again, we have renamed these probes throughout the manuscript to “telomere 1”, “middle”, or “telomere 2”, and we adjusted the cartoons in new Figure 5.

10) Line 268-269: Explain how the whole mounts validate the findings. The images are not as clear. What is the significance of the apyrene nuclei? More description of how these images are important to the paper would help.

We appreciate this suggestion. We have added a sentence about the benefits of whole mount versus chromosome spread approaches (lines 286-288) and have included the significance of apyrene nuclei (line 293-294).

11) Figure 5: The authors conclude the leptotene homologs not paired. How is this defined and measured and is it 100%? Some quantification is need because some nuclei seem to show some pairing.

This is important because it indicates the state of the chromosomes before SC assembles. Conceivably this could be measured (eg the distance between each homolog).

For all our analyses, we classified cells with unpaired but linear chromosomes as leptotene/zygotene (as well as based on the DAPI stain being a dense lampbrush structure), and we have clarified this point in the main text (lines 175-176) as well as in the methods section. Quantification of pairing as we understand the reviewer to be suggesting is not feasible in the whole-mount images due to the close proximity and overlap between neighboring cells. However, we have further quantified pairing and partial pairing in zygotene cells from testes squashes in new Figures 4 and 5, and we hope this has addressed the reviewer's concern.

12) Line 312 – define "polytrophic meroistic"

We have added the definition, lines 330-331.

13) Lines 311-312 and 326: Is it surprising that chromosome-wide pairing persists in females? Do the cited references have data to suggest they should not be paired along their length? A review from von Wettstein (Ann Rev Genetics 1984) suggests the female meiotic chromosomes remain paired along their entire length by a modified SC structure. This analysis would also benefit from some quantitation to know if there is any variation. Because the chromosomes are small, this would help the conclusion that the chromosomes are paired.

We thank the reviewer for posing this question. Based on the existing literature, including the von Wettstein text, we believe that while homologs should indeed be held together by the modified SC, the modified SC should be large enough to create separation between the homologs in the mature larval oocytes. To better understand pairing in female ovaries, we performed super resolution imaging and indeed can detect a space between the homologs. Furthermore, the distance between homologs gradually increases throughout oocyte maturation (new Figure 7). We hope these new data and our explanation here satisfy the reviewer's concern.

14) Line 357: In most organisms that SC components are poorly conserved. In mammals there are SC components with no known paralogs in other organisms. Therefore, Drosophila is not that unusual, and indeed C(3)G is a ortholog of SC proteins in other organisms.

We have removed this sentence about SC conservation in *Bombyx* versus flies.

Reviewer #3:
In this manuscript, Rosin et al. employ Oligopaint DNA FISH to examine homolog pairing and compaction during both male and female meiosis in the holocentric Bombyx mori. This study shows Oligopaints can be used in both squashed and whole tissue with high specificity in this silkworm moth. It also reveals that telomeric regions face poleward, likely acting as localized kinetochores, with either telomere having an equal probability of facing poleward and potentially explaining how these chromosomes segregate in the absence of CENP-A. This is a very interesting study that shows the use of Oligopaints for the first time to assess compaction and pairing of multiple, whole chromosomes in both male and female meiosis and is the first study of this kind in Lepidoptera, opening up various avenues for future discoveries about meiosis in B. mori. This is a well-written manuscript with beautiful images and striking observations that showcases the importance of looking at meiosis in different species and will be of interest to the broad

readership of PLoS Genetics. Below are some questions and recommended revisions:

Major:

1- Page 6, Line 117: Please add a few words in the main text explaining the rationale behind the choice of the six autosomes and the Z sex chromosome being targeted in this analysis (i.e. were the six autosomes selected at random or for specific reasons from among the 27 autosomes in this organism? The rationale for why the W chromosome is not targeted is mentioned in the legend for Fig S5, but it would be helpful to raise this point up front in the main text when describing why the Z is targeted).

We have added our rationale for chromosome choice to the main text (lines 130-132) as well as the rationale for omission of the W chromosome (lines 136-138).

2- Page 8: The authors report noticing that all chromosomes do not pair simultaneously during early meiotic prophase I in larval testes squashes. Are there specific chromosomes among those assessed that are achieving pairing always earlier or later? Is there something inherently unique about these if that is the case (i.e. chromosome size, enrichment for germline-expressed genes, heterochromatin/euchromatin distribution, faster/slower replication prior to meiotic entry…)?

We thank the reviewer for this suggestion, and we have more thoroughly quantified pairing in zygotene (new Figures 4 and 5) and discussed these findings in the main text (beginning in line 216, as well as in the discussion, beginning in line 373). Interestingly, our results show that gene-rich telomere domains within chromosomes more often pair first and additionally that large, gene-rich chromosomes pair first.

3- The authors should indicate the frequency with which they observed the large chromosome loops (Fig 3A) and what they think they represent? Are these indicating that pairing does not start at a single region or chromosome end like in worms, but instead at multiple regions between the homologs (or at opposite ends of the chromosomes?)? Can the authors correlate this with either how SC assembly unfolds in this organism (i.e. progressively from one end or from multiple nucleation sites throughout the chromosomes) or location of double-strand break sites?

While we cannot correlate our pairing configurations with SC assembly (due to lack of antibodies), we have better quantified pairing initiation as described in our above response. We have also added some speculation about what these partially paired structures might represent to the discussion (lines 376-385).

4- Please move Figure S8 into main Fig 5 since this will be very helpful for those not familiar with staging in the testicular lobe in Bombyx mori. This will also be consistent with what was done in Fig 6 by including a useful schematic of the 5th instar larval ovary in panel A.

We thank the reviewer for this suggestion. We have made old Figure S8 panel A in new Figure 6.

5- Fig 5 C and D- There seems to be a comet tail signal revealed with S2 and S3 on metaphase I chromosomes. Can the authors briefly explain this either in the main text or figure legend?

The reviewer is correct. However, this observation is not biologically significant and is merely excess background signal due to the nature of whole mount imaging and the thickness of Bombyx testes. We

have added a comment to the figure legend in order to address this point (lines 552-554).

Minor:
1- Reference #40 is duplicated as #45. *And* 3- Page 6, Line 123- Reference is cited instead of reference #.

Thank you for pointing these out. We have corrected the references.

2- Page 4, Line 86- Typo (word missing) in "for segregation meiotic chromosomes…"

We have corrected this sentence.

4- Fig S2: Please use colored arrows or arrows and arrowheads to indicate the mitotic and meiotic clusters.

We have added colored arrows to Figure S2 per the reviewer's suggestion.

5- Figure 2 - legend indicates "D, larval ovary", but panel D is labeled as Testes in the figure. Also, correct where it says "metaphase I (F) cells" to (G) instead? *And* 7- Figure legend for Fig S7- Typo "in the this cell line".

Thank you, these errors have been corrected.

6- Please indicate number of larvae examined in the legend for each figure.

We have added "n" values to all appropiate figure legends.

Reviewer #4:
In their manuscript, Rosin and colleagues analyzed the pairing of maternal and paternal chromosomes during the first steps of meiosis in Bombyx mori. They used thousands of fluorescent oligos to label whole chromosomes or specific subregions of single chromosomes. With these tools, they analyzed chromosome behavior at each step of meiotic prophase I on fixed germ cells in male and female silkworm. The images are stunning. I don't have any major criticism. Obviously, this study is descriptive by nature, but it sets the stage for future functional studies. It will be a reference in this field.

My comments are rather curiosity questions:
1) Do chromosome start to pair by the same regions?

From our new quantification (added to new Figure 4 and 5), pairing is almost always initiated at telomere domains, with some chromosomes showing bias for one telomere over the other. We have also included a brief discussion of "pairing" as measured by Oligopaint colocalization versus true synapsis as well as the possibility that SC formation is not completely correlated.

2) Is there an order in chromosome pairing?

There is not a discrete order for pairing, but our quantification (again in new Figures 4 and 5) suggests that larger, gene-rich chromosomes more often pair earlier.

3) When are double-strand breaks formed compared to synapsis? Is it possible to perform double-labelling with other meiotic landmarks? Such as the synaptonemal complex or DSBs?

This experiment is theoretically feasible, and we and others are working on generating custom antibodies to better study the SC and DSBs in *Bombyx* (which currently do not exist) to answer such questions in the future.

Minor comments:
1) I would add more introductory material. Especially, regarding the C. elegans literature, which may not be obvious to every reader.

We thank this reviewer for the suggestion, and we have added more introduction regarding *C. elegans* literature to the introduction and throughout the manuscript where relevant.
